# A transitive perspective on the relief of psychosomatic symptoms

**DOI:** 10.3389/fpsyg.2022.821566

**Published:** 2022-10-14

**Authors:** Walter Tschugguel

**Affiliations:** Department of Obstetrics and Gynecology, Medical University of Vienna, Vienna, Austria

**Keywords:** process concept, psychosomatic disorders, symmetry breaking, Jung-Pauli collaboration, spatiotemporal neuroscience, symptom prescription

## Abstract

A key element of successful psychotherapy for the treatment of psychosomatic disorders is that patients recognize and change the meaning of their experiences. Such changes are brought about by appropriate verbal referencing of symptoms currently experienced within a given narrative. The present theoretical paper argues that changes are not based on better, more adaptive narratives *per se*, but on the transition (or linkage) process itself that is experienced between different narratives. This view is theoretically justified in various ways: first, it is accounted for through contemporary spatiotemporal neuroscience, which aims to connect mental and structural aspects *via* a common dynamic property or, according to Northoff, the “common currency” of a brain’s orientation along its embeddedness in its contextual world, i.e., body and environment. Second, it is justified through the physics concept of “spontaneous symmetry breaking,” which is used analogously to “suffering from symptoms.” If the sufferer is willing to experience a process of “going back,” that is, moving away from the previous narrative (or aspect) by verbally relating to the felt aspects of the symptom in question (i.e., approaching its meaning), they are moving toward symmetry or an underlying dynamic alignment with their world context. Clinical predictions are derived from the theoretical arguments.

## Introduction

Alliance, expectations, and empathy have been denominated as common factors of therapeutic effectiveness for different psychotherapeutic contexts (Wampold and Imel, 2015). Moreover, all psychotherapeutic schools agree that transforming the meaning of experiences is essential for recovery (Frank, 1986). It has recently been suggested that joint patient–therapist interactions can produce meaningful transformation through the patient experiencing novel, adaptive narratives (Locher et al., 2019; Sensky, 2020). This has been exemplified based on three different and commonly used psychotherapeutic approaches (Locher et al., 2019), through which the patient and therapist co-construct a meaning of the patient’s current reality representation, that is, their illness narrative. From this shared ground, a process of meaning transformation enables a novel narrative that is more plausible (cognitive therapy), more functional (systemic therapy), or more congruent with the patients’ self-concept (person-centered therapy) (Locher et al., 2019). However, it remains unclear whether meaningful transformation signifies the approximation of an existing, non-adaptive narrative (or system of meaning) to a new, more functional, and adaptive one (metaphorically, comparable to arriving at a “better-adapted” train station), or whether the experience of the transition itself (metaphorically, the train journey itself) is crucial for relief.

To answer this question, I aim to derive arguments from empirical data, which show that it is not the achievement of adaptive concept forms, that is, “adaptive narratives” *per se* that is effective, even if these narratives elicit newer and more advantageous meanings; rather, it is the transitive (or linkage) process between the two substantive concept forms itself that is beneficial. Arguments to this end are obtained from the psychotherapist Eugene Gendlin, modern physics, and the Jung-Pauli conjecture, considering the contemporary spatiotemporal neuroscience perspective (references given below). Theoretically grounded recommendations for clinical use are derived from the presented arguments.

## Evidence for the efficacy of symptom prescription in psychosomatic patients and the rationale for studying a patient treated with this method to test the argument in question

To investigate the research question concerning the primary relevance of the transition process between narratives in psychotherapy—as outlined above—three premises are required: the choice of (i) a suitable patient, (ii) an intervention at the subject level of the patient and (iii) the patient’s willingness to engage in the therapy process.

I firstly focus on a case selected from a group of patients known to be unable of verbally expressing their emotional status, whilst still experiencing the somatic component related to their affective reaction (summarized in de Greck et al., 2013). These patients are respectively referred to as suffering from psychosomatic (or somatoform) disorders. Several psychotherapeutic approaches have been applied to these patients such as psychoeducational interventions, stress management procedures, cognitive-behavioral therapy, brief dynamic therapy, family therapy, group interventions (reviewed in Fava et al., 2017), psychodynamic psychotherapy (Bronstein, 2011; de Greck et al., 2013), or clinical hypnosis (Wilkinson, 1981; Tschugguel and Tschugguel, 2010; Häuser et al., 2016).

Second, I describe the treatment of such a patient by means of symptom prescription for the following reason:

By definition, a symptom is defined by the patient as a behavior that is uncontrollable, involuntary, and spontaneous (Weeks, 2013). Prescription (or reframing, decontextualization) of symptoms is a technique that has been proposed as the single common denominator of efficacy for all systems of psychotherapy (Weeks, 2013). The patient must alter his or her attitude toward a behavior if it is allowed expression during joint patient-therapist interaction. “When a client is able to change the context of the symptom, the meaning inevitably changes only because the client is able to demonstrate some control over the uncontrollable, some volition over the involuntary, and some mindfulness over the spontaneous (mindless or automatic behavior)” (Weeks, 2013).

The efficacy of symptom prescription in a narrow sense has been demonstrated by effectively influencing symptoms and their persistence in a control group study involving patients with multiple, chronic, medically unexplained physical symptoms and severe physical illness (Schwarz et al., 2016), a meta-analysis (Kern, 1993), and a control group study of socially phobic students (Akillas and Efran, 1995). Its core concept is a type of “paradoxical intervention.” As early as in 1977, the “paradox” of “taking control by giving it away” was suggested as the common element of all psychotherapies (Weeks, 2013). According to Lankton and Lankton (2013), who described paradoxical treatment in Ericksonian hypnotherapy, “it symbolizes the natural wisdom of impermanence,” that is, the transitive experience. The patient is asked to produce their symptom precisely, but now as part of the therapy in terms of a desirable process to gain self- and mutual control (Weeks, 2013), in the Aristotelian *sensu causa finalis*. This helps the patients to change their perspective in such a way that these changes are easier to make, referred to as “reframing” by Paul Watzlawick (Weeks, 2013). Starting from Lankton’s view that “… the key is in the process interface between therapist and client. It’s in that in-between. You can only create something at the interface” (Watson, 2013), I place further emphasis on the process interface. However, any attempts to understand what a “process interface” is immediately run into an intricate ontological enigma. The core of this enigma lies in the question of how an intervention allowing a patient the fullest expression of freedom in developing a new frame of reference (Weeks, 2013), attributes change to themself. According to Hameroff and Penrose (2014), it must be “a non-computable factor,” independent from a “neurocomputational approach to volition, where algorithmic computation completely determines all thought processes, [and] appears to preclude any possibility for independent causal agency, or free will. Something else is needed.”

Contemporary clinical hypnosis has been used to prove the concept as a suitable type of intervention at the patient. Hypnosis is a therapeutic procedure clinically effective in a wealth of psychosomatic conditions (reviewed in Häuser et al., 2016). It is defined as both an altered state of conscious awareness and a procedure to induce such a state (Peter, 2015; reviewed in Häuser et al., 2016). Once induced, physiological, cognitive, affective processes, and behavior are under disposition. The hypnotic trance state can be induced either by the therapist or alone (self-hypnosis) and is distinguishable from other states of consciousness (i.e., waking state, sleep, meditation) according to electroencephalography and imaging methods. The characteristic features comprise altered time perception, selective amnesia, age regression, marked inward attention, and reactions to suggestions. Contemporary clinical hypnosis is a non-authoritarian, resource- and solution-oriented method, in which the focus is on the patient’s own potentials (Peter, 2015; reviewed in Häuser et al., 2016). Therefore, patients should be informed that hypnosis is not a condition that the hypnotist induces in them, since this would preclude free will. Rather, it is a condition that occurs within the patient naturally, in a proper atmosphere created by the therapist, like self-hypnosis, which occurs automatically during various everyday situations, such as driving a car (Tschugguel and Berga, 2003). To induce hypnosis, verbal, or non-verbal suggestions (e.g., visual signals given through the finger) are provided in such a way that they merely serve as a proposal (Peter, 2015; reviewed in Häuser et al., 2016), comparable to inviting the patient of entering a supermarket of possibilities, where they are now free to choose what to grab.

These three initial premises serve to test the theory of the primary relevance of transitive experience: (i) physical symptoms on the one hand vs. inability to verbalize the underlying emotional state on the other, (ii) willingness to enter a state of dispositional affect response—such as that of hypnosis—based on trustful cooperation with the therapist, and (iii) success in generating the symptom under controlled conditions with the therapist.

## Gendlin’s theory of process concepts

Some useful insights into the “process interface” and transitive experience have been provided by the philosopher and psychotherapist, Eugene Gendlin, who proposed a concept to overcome the traditional “hard problem of consciousness,” that is, the irreconcilable dualization of the psyche and body, describing them as “imprecise, first-person involving” and “mechanistic, third-person space time-grid precise assumptions,” respectively (Gendlin, 2000). To overcome the dualizing split, he introduced a third factor, the “process concept.” His allegory essentially explained the following (modified from Gendlin, 1964, p. 18): if an animal were hungry, it would normally symbolize this by eating and continuing its organismic digestion process, that is, it would refer adequately to its feeling of hunger. Conversely, if it had been trained to ignore its experience of hunger (or illness), it would bite itself in the leg. This is exactly the case with human feelings of guilt, shame, or wickedness that occur “as if” they are reactions to feelings, due to our habit of not adequately referencing the underlying feelings. Gendlin concluded that the unconscious involved an incomplete process, a “convention” of “muscular and visceral blockage,” excluding experiences from awareness. It must be acknowledged, however, that psychosocial stress paradigms are by their very nature human. However, the use of Gendlin’s allegory includes the non-social stress paradigms that also exist in animals. They currently serve as animal models for psychoneuroimmunology understanding of the etiology of psychiatric and somatic diseases (Reber and Slattery, 2016). The “blockage” assumption matches a very early notion of the philosopher Arthur Schopenhauer, who described “body blockage” in the following terms: “the body is deceived in ‘good faith’ but under ‘false pretenses”’ (Schopenhauer, 1859/1977).

This raises the question: what can encourage the sufferer to refer to their bodily experiences to unblock implicit feelings, serving to alleviate symptoms? In the words of Fava et al. (2019), how can suffering “become the sources of positive insights” as a “prelude to desirable adaptive changes”?

Gendlin arrived at an answer: to reactivate the completion of this process, that is, unblocking, he proposed a “law of the reconstitution of the experiencing process: When certain implicitly functioning aspects of experiencing are carried forward by symbols or events, the resulting experiencing always involves other sometimes newly reconstituted aspects, which thereby come to be in process and function implicitly in that experiencing.” (Gendlin, 1964). I refer to this in the later sections of the paper, following the presentation of a case.

## Experiencing symptom prescription: A case

In what follows, a succinct core of symptom prescribing is illustrated using a cascade of interactions from an actual case of the author’s office. The patient (P) was defamiliarized by removing her name, age, and other details of her history to retain her anonymity, the author was regularly P’s therapist (denominated here as operator O) from February 2018 until November 2019; the session presented in subsequent paragraphs took place in the initial phase of therapy. The therapy consisted of a total of 22 sessions, initially monthly from February to September 2018, then twice a month from October 2018 to February 2019. There were no sessions from February to October 2019 because the patient was abroad. Between October and November 2019, eight weekly sessions took place. Then, the patient had to go abroad again. Subsequently, there have been no further sessions so far.

To understand the connection between theory and practical procedure, the theoretical concept behind the procedure is briefly presented. To this end, the core of case description is added in the light of P’s previous findings through O’s questions and Ps answers regarding the possible meaning of the prescribed symptoms in the form of experiences reported by the patient during her trance. Strictest attention was paid on O’s part not to make any judgmental comments and only to maintain the flow of P’s experience.

The relationship between P’s previous insights, her present experience during trance, and O’s attitude of only maintaining the flow of her experience during trance can be seen as P’s key to understanding future challenging experiences in a new light, being recontextualized by means of the surprising, shared experiences in trance.

### Theoretical concept of procedure

As already described by Langewitz (2011), a careful and detailed history sampling of P is necessary. In doing so, one should pay less attention to the complete recording of P’s biography than to an understanding of very specific, concrete experiences, which are clearly affectively connoted by P, and which can be asked about. It is essential for O not to ascribe any generalized meanings to this experience explicitly or implicitly, but rather to ask about the concrete meaning of this situation and the actions associated with it. In other words, we inquire about the concrete beyond the empirical that might not yet be necessarily knowable to P, since she always constitutes her past from present meanings (Sartre, 1943/1956 transl. by H. E. Barnes, p. 563).

Hence, the commonality of various empirical drives of P is what Sartre denominates as the “empirical attitude (of P herself as) the expression of choice of an intelligible character,” namely, her “fundamental meaning” (Sartre, 1943/1956, p. 564). This fundamental meaning can now be revealed by “deciphering the meanings of the person’s being-in-the-world” (Sartre, 1943/1956, p. 564), that is, P’s being-in-concrete-situations. As such, therapy’s “point of departure is experience” (Sartre, 1943/1956, p. 568).

From this moment on, one must always be prepared for the fact that the symbols chosen by P can already change their meaning in P (and usually do, insofar as space and time for a possible flow of experience on the part of P are unfolded by O during preliminary conversation. Subsequently, the reader can see that the theory of experiential thoughts’ transition takes root in all phases of the therapeutic process). Only the grasping of individual, momentary phenomena, no matter how minimal they may be, enables their grasping in the conceptual (e.g., “I still pay so much attention to my parents getting along! Yet, I have a family myself!”). It is exactly at this point that the implicit meaning of the symptom becomes explicit and, thus, dispensable. The possible meaning assignments of the symptom run out of further words; P begins to discover that she must no longer let herself be “driven” by her symptom. Therefore, she can now discover new degrees of freedom of action. Once P has become dispositional with respect to the assignment of meaning to her symptom, the trance experience can envelop this process of disposability with an alternative experiential context. Here, we come to hypnosis, a mutually agreed process of inner focusing that opens P’s personal space and time to experience a change in the sub-modalities (i.e., quality, extent, intensity, weight, etc.) of the symptom prescribed to her, from the context of the previous conversation.

### Application of concept during therapy sessions with P

The therapy session conducted with P is described here. During the initial session, P’s history was taken. She grew up abroad and moved to Europe with her family over 10 years ago. She presented with a diagnosis of *“longstanding irritable bowel syndrome”* and *“suspected food allergies that have never been measurably verified,”* combined with recurrent newly emerging episodes of *tinnitus*, experienced as *“light and tingling sensations that started a few months ago,”* associated with *“fear of losing control in challenging situations.”* At the end of a detailed, 2-h anamnesis, P and O concluded that the fundamental meaning of P’s unconscious bodily process since her earliest childhood was to essentially act as a mediator between her polyamorous father and her mother’s saintly figure to keep their marriage going. She continued her expert role in her profession as a mediator. She never learned to sense, recognize, or appreciate her own physical and personal boundaries. Her mother, who suffered several early miscarriages before P was born, was always caring, quiet, and reserved in P’s memory, accepting the role assigned to her by tradition, and died of ovarian cancer a few years earlier. From then on, P’s father required P to take care of him, as tradition demands of the women within the family.

Multiple times during her adolescence, P asked her mother how she put up with her father’s charming behavior and received answers such as “Oh, let him.” P repeatedly felt a tightness in her head, neck, and shoulders, and sometimes suffered from shortness of breath associated with now diagnosed gluten intolerance. Food always got stuck in her, combined with a feeling of fullness and the general impression that “once everything is in the cells, it does not want to come out any time soon.” As a child, she often felt the need to hide in her father’s wardrobe, a small, dark room where she could “hear” the “sound of silence,” completely undisturbed. The allergies kept changing, with an allergy to food turned into an allergy to pollen, and stress aggravating the symptoms. Toward the end of the initial session, P was informed about hypnotic trance and its corresponding phenomena and was casually asked if she wanted to experience it for a few more minutes before coming back. She was very happy to do so, with O’s appropriate invitation already being delivered slowly. During this brief 10-min trance, she was invited to experience autonomic ideomotor limb movements (twitches and arm raises) of early childhood that were familiar to her body from her memory, with the accompanying comment of “being perceived by everyone else as an autonomous human being with boundaries.” In other words, a reference to her childhood ability of setting *autonomous behavior*, and thus her *dignity of “being human”* and not a “heteronomous function of other people’s needs,” was implicitly emphasized. In the next session, which took place about 7 weeks after the first one, P reported that she was already feeling better. She cried that her father always presented her as a “decorated little horse.” During this recollection, she felt pressure in her throat and a feeling of “thickening of breath” coupled with the account of her dreams, in which she could neither speak nor scream and her voice could not “get out” of her gullet. With the instruction to continue from this topic the next time, symptoms currently arising in the context of her memories disappeared. In the next session, she reported that when she was overwhelmed at work, during which she would function like a robot, and she would always have a similar dream at night. The physical overload caused by exceeding her stress limit often triggered violent anger and abdominal pain in her; she looked in the toilet mirror at work and said to herself, “that is not you!” Thus, she was asked by O whether she would like to “remember this sort of gut feeling” that she just talked about during trance “to make new discoveries from there.” With her consent obtained, she was told that she is not alone with her feelings, is in a safe environment, and O can be trusted. When P was in a trance with her eyes closed, O triggered a memory by asking P what she wants to start remembering first (i.e., using Ericksonian trance-inducing double binding; Erickson and Rossi, 1975). “Is it a picture, a sound, a feeling, a smell, or a taste?” (i.e., requesting sub-modalities to trigger the memory). She did not have to answer; it was just a matter of finding out how it made her feel, and when she was ready to rehearse, she could confirm this to O by, for example, moving her left index finger (ideomotor activity as evidence of intrinsic activity). Once she moved her finger, O asked, “What is it now?”; P responded, “pressure in the belly.” Then, O replied, “Please try to make the feeling stronger, let time slow down until it almost stops. Your space becomes wide, wider, and wider, so that you can see very far, very comfortably into the inner vastness. (Transitive state of *“slowing down of time and expansion of space*”) [Pause for several seconds] What happens now?” P said, “It has moved upward, suddenly in the throat” [P is choking now]. O replied, “Please hold on, take your time, I am with you, and you are safe, wait and see what comes, slowly but surely.” P stopped choking and sat in amazement. O asked, “What is it now?” P said, “I see a tall woman with long black hair, a white face, her hair fluttering in the air, floating above the ground.” [P was crying at this moment]. O responded, “Take your time, that is right, it is good to let it all come, more and more, just let it come, let it get stronger and stronger” [pause, no further speaking]. As soon as O saw her body relaxing, he invited her to dehypnotize with the instruction, “Remember any experience that your subconscious mind wants you to remember now after you wake up. And once you are fully awake, please stretch your whole body so that you feel as relaxed, fresh, and strong again as if you had slept well all night” [pause]. After waking up, P said, “Crazy, that was intense!” O asked, “What do you mean, pleasant or unpleasant, or unclassifiable, but just intense?” P replied, “Violent, yes, enormous, strange” [pause, as P was still completely preoccupied with her feelings]. O said, “Your body will surprise you now every other day, almost imperceptibly, [pause] as if your perspective on all events changes, especially on all challenges [pause] that you have experienced again and again in the past, with—little—changed perspective [pause]. At the same time, your perspective has changed by 0.347 or 0.591 degrees [a metaphor for arbitrarily low numbers that gift-wrap the mediated notion of minute and therefore initially imperceptible changes], and a little more each day [pause].” As soon as P began to smile (which is often the case after such a procedure), O asked, “What are you going to do today?” This question typically induced amnesia to distract P from the previous trance, bring her completely out of trance, and make her feel like everything is done for today, that she can now go about her daily activities with peace of mind. The next session was scheduled 4 weeks later because, in O’s experience, reflections on the perceived changes need some configuration time to emerge fully. At the next meeting a month later, P reported that a lot had happened. Enormous anger, but also strength, emerged in her. She had outbursts of anger in many situations, but also had corresponding feelings of guilt. She must now learn to be careful with the boundaries she feels, so as not to hurt anyone. She was also suddenly able to distinguish mendacity and feigned kindness from genuine kindness, which gave her considerable symptom relief. Stomach pain and sleeping problems (which she initially only mentioned in passing) were no longer an issue, and her tinnitus had improved by around 40%. In further sessions, her constantly new discoveries in dealing with herself and topics such as her father’s death that had taken place in the meantime, were taken up. These investigations were conducted during trance or conventional exploration, and were integrated into P’s current horizon of experience, according to her directly conveyed images or feelings that accompanied these themes. Ultimately—according to the last meeting in November 2019—P found that any food, “even foods high in histamine, such as eggplant or radishes,” was tolerable. Her breathing also improved, allowing her to breathe much deeper. She effortlessly distanced herself from her family problems and could now concentrate much better on her further plans. Stressful statements that she had learned in her childhood, such as “Nothing is free in life, my child” or “You have to pay for everything in life, be it freedom, happiness or ultimately even with life,” were now regarded as attitudes or points of view that did not belong to her and had very limited meaning. In accordance with the previous considerations, this brief example shows that the sufferer P experiences herself in a position where she can find verbal symbolizations and events related to her suffering. This has previously been described based on typical cases of somatoform “chronic pelvic pain disorder” (Tschugguel and Hunter, 2008; Tschugguel, 2018).

At what point in the contention between P and O does the transitive state unfold? Since the transitive state is a purely subjective quality, the sequences of the process from which O selects a particular one for the following description are purely subjective, that is, they do not necessarily coincide spatiotemporally with those that P experiences. O can never know when and where P experiences transitions. This spatiotemporal non-correspondence of events is the essence of P and O’s joint engagement. It is precisely because of this empirical indeterminacy that the sufficient condition of pure quality is given, that is, therapeutic impulse in its narrowest sense. In the case described here, O experienced a transitive state after having pronounced *“slowing down of time and expansion of space”* in that O has perceived atmospherically between himself and P a change.

This transitive state sequence, *“slowing down of time and expansion of space,”* is explained in the following from perspectives of physics, philosophy, and neuroscience.

## Inferring a theory from the case based on physics, philosophy, and neuroscience

### Symmetry breaking

A very useful concept is that of symmetry and symmetry breaking by physicist Wolfgang Pauli, which has already been used by Atmanspacher and Primas (2006) to fundamentally investigate the relationship between mind and matter from a physical point of view. According to them, “symmetry” is one of the “empirically inaccessible,” presuppositionless “first principles of physics,” and is “defined as invariance under a certain set of transformations.” They give an example: “The laws of physics treat all directions of space as equivalent, but in daily life there is a crucial difference between horizontal and vertical directions.” Analogously, “symmetries” (corresponding to the principle of equivalence of all directions in space) “are never empirically attainable; they can only be theoretically derived [based on] phenomena that exist due to broken symmetries” (corresponding to the concrete, empirical observations of horizontal and vertical directions in the space of our daily life). The fact that observed phenomena generally do not have the symmetries of the laws governing them was clearly recognized by Pierre Curie (1894): “Asymmetry is what creates a phenomenon.” In a perfectly symmetrical situation, there are no distinctions, so reality does not appear in structured form” (Atmanspacher and Primas, 2006, p. 3). Accordingly, Pauli further mentions, “It would be most satisfactory if physics and psyche could be conceived as complementary aspects of one and the same reality, which, (in itself), “is not directly accessible.” Analogous to Pauli’s concept of complementarity of mental and physical phenomena is the blocking of principally unobservable, unblocked, theoretically healthy physical interactions and experiences into empirically observable, asymmetrical, pathological aspects of mental (e.g., *“fear of losing control”*) and physical issues (e.g., *“irritable bowel syndrome”*). This analogy follows the physical concept of “symmetry breaking” from a theoretically possible, healthy state of symmetry (*autonomous behavior*, *dignity of “being human”*) into pathological, observable and, hence, asymmetrical aspects of psyche (*“fear of losing control”*) and body (*“irritable bowel syndrome”*). Subsequent predictions about these henceforth denoted observable aspects (phenomena) are deductible. On the one hand, these are subjectively experienced bodily sensations, in the form of symptoms. On the other hand, they occur together with measurable, structurally observable variables (e.g., patterns of functional magnetic resonance imaging signaling, electroencephalogram, or changes in heart rate, blood pressure, skin conductance; whatever the clinical circumstances in question are). These symptoms only become obsolete once the sufferer begins to appropriately reference them verbally as “vehicle(s) of meaning” (according to Langer, 1978, p. 52, quoted by Atmanspacher and Primas, 2006) (as illustrated in the case above). However, how this obsolescence occurs requires further theoretical elaboration.

### According to which hypothetical principle could symptoms become obsolete if they are referenced verbally by the sufferer, i.e., symbolized?

#### The Jung-Pauli collaboration and contemporary spatiotemporal neuroscience

The “Jung-Pauli collaboration” is a paradigm, which is very useful in explaining such psychophysical correspondence effects: “… [Wolfgang] Pauli and [Carl Gustav] Jung proposed the idea of psychophysical correspondences (‘synchronicities’) between psychological and physical subdomains of an underlying hypothetical background reality” (Atmanspacher and Fach, 2005, p. 202). In a letter to Jung, Pauli originally wrote (translated from German by the author):

“Whether one speaks of the ‘participation of natural things in ideas’ or of a ‘behavior of the metaphysical, that is, of things that are in themselves real,’ the relationship between sensory perception and idea remains a consequence of the fact that both the soul of those who know as well as the objects recognized in perception are subject to an objectively conceived order. Any partial knowledge of this order in nature leads to the formulation of statements that on the one hand concern the world of phenomena and on the other hand also use general logical concepts in an idealizing way. The process of understanding nature, as well as the happiness a person feels in understanding, that is, when he becomes aware of new knowledge, seems to be based on the meeting of already existing internal images of the human psyche with external objects and their behavior. As is well known, this conception of natural knowledge goes back to Plato and is also very clearly represented by Kepler” (Pauli, 1948/1992).

In summary, Pauli assumed that an underlying, however undefinable, and unapproachable, ontic background realm that) grounds all aspects (*“fear of losing control,” “irritable bowel syndrome”*) of being a human. This ontic background realm corresponds to the Kantian “thing (or object)-in-itself.” According to Kant,

“Space and time are its [i.e., the object’s] pure forms, sensation in general, its matter. We can cognize only the former *a priori*, i.e., prior to all actual perception, and they are therefore called pure intuition; the latter, however, is that in our cognition that is responsible for it being called *a posteriori* cognition, i.e., empirical intuition. The former adheres to our sensibility necessarily, whatever sort of sensations we may have;” (*“slowing down of time and expansion of space”*) “the latter can be very different.” (*“Fear of losing control,” “irritable bowel syndrome”*) “Even if we could bring this intuition of ours to the highest degree of distinctness, we would not thereby come any closer to the constitution of objects in themselves. For in any case, we would still completely cognize only our own way of intuiting, that is, our sensibility, and this always only under the conditions originally depending on the subject, space, and time; what the objects may be in themselves would still never be known through the most enlightened cognition of their appearance, which is alone given to us” (Kant, 1787/1998).

However, are we really compelled to describe this background realm as ontic, that is, empirically inaccessible, since Kant himself claims some outer given “sensation,” which affects our intuition to form any object? Attempts to shed light on it have been put forward by Karl Friston (2010), who conceived “the brain as a generative model of the world it inhabits,” due to the brain’s capacity to minimize its energy consumption by minimizing errors in predicting sensory information. According to Friston, by tracing internal mental and external neural spheres back to the same underlying constitutional process, dualism is not even a possibility, let alone a reality (as described in Northoff, 2014). That is, the patient is offered a way to autonomous behavior and thus to her dignity of “being human.” In this sense, the notion of an empirically inaccessible background realm dissolves. This view corresponds to the work of Georg Northoff who, from evidence of spontaneous (or baseline, inactive, background, non-task induced) spatiotemporal dynamics of brain activity without any brain function, as seen, for example, in anesthesia, suggested that baseline, spontaneous, spatiotemporally orchestrated brain activity is the neural predisposition of all stimulus- or task-induced activity (Northoff, 2012, 2014; Han et al., 2013; Northoff et al., 2020) (*“slowing down of time and expansion of space”*). This view is summarized as follows:

“Information processing is no longer regarded as [the] primary purpose of the brain’s activity, as it is replaced by the brain’s capacity to transform and integrate different temporal and spatial scales of brain, body, and environment. One example of that is consciousness that may consist in exactly that, the transformation of different temporo-spatial relations into mental features—this is well compatible with the leading theories of consciousness like the Global Neuronal Workspace Theory, the Integrated Information Theory, and especially the Temporo-Spatial Theory of Consciousness” (Northoff et al., 2020).

Consistent with this argument, Northoff concluded that, according to the paradigm of spatiotemporal neuroscience, the mind-brain relationship is less important than the world-brain relationship, owing to the dynamic spatiotemporal alignment of the brain “along” the world (Northoff, 2012), that is, its sociocultural context (Han et al., 2013). Northoff explained this view in his early 2012 paper:

“…The brain shows neural activity generated by itself, independently of the stimulus it encounters. This spontaneous or intrinsic activity is described in operational terms as resting-state activity. What neuroscientists observe as stimulus-induced activity is a mixture of both the brain’s intrinsic activity and the neural activity changes related to the stimulus. Consciousness and self are consequently assumed to be predisposed by the brain’s intrinsic activity (i.e., resting state activity) and become manifest during the resting state’s modulation by extrinsic stimuli from body and environment” (Northoff, 2012, p. 357).

This contemporary view of brain function (Northoff, 2012, 2018; Singer, 2013; Han et al., 2013; Northoff et al., 2020), has been denominated the “Kantian brain” (Fazelpour and Thompson, 2015). However, contradicting Kant’s view, which is based on the idea of the stimulus as an empirically unattainable “thing-in-itself,” Northoff emphasized the embeddedness of the brain in its sociocultural and body context. By such conceptual replacement of the “ontic domain,” as previously described in the Jung-Pauli conjecture, with an “empirically accessible” one, that is, an *a posteriori* connection of brain and psyche (Northoff, 2018) by means of the “sociocultural (or world) and body context” as a “common currency” (that is, the patient is offered a way to autonomous behavior and thus to her dignity of “being human.”), Northoff established spatiotemporal neuroscience as a scientific paradigm. In accordance with the latter perspective of the resting-state alignment of the brain along its world and body context, contents of suffering can symbolize the sufferer’s inability to dynamically (or flexibly) align to their changing, sociocultural world and body context. Verbally designated symbols of the sufferer’s experience, for example, *“fear of losing control*” and *“irritable bowel syndrome,”* as seen in the present case description, may thence be understood as structurally representable subdomains; they may be considered “aspects” of such dysfunctionality within a given world and body context, for example, “having learned to act as a mediator between her parents to keep their marriage going without feeling, recognizing, or appreciating her own physical and personal boundaries.”

#### How is the term “aspect” used here?

Since a person’s suffering consists of contributions from various aspects (*e.g., “irritable bowel syndrome, suspected food allergies that have never been measurably verified*, combined with *recurrent newly emerging episodes of tinnitus”* on the one hand, and *“fear of losing control in challenging situations”* on the other hand) of their experience of illness (Sensky, 2020), I will now explain what I imply by “aspect” in this regard. William James described the thought passage (or linkage phase) between stable mental representations, that is, contents of consciousness (designated by him as “substantive states,” e.g., inner images of symbols), as the “transitive state” (e.g., *“slowing down of time and expansion of space”*):

“Transitive states [are] places of flight [of] the transitive parts of the stream of thought. It then appears that the main end of our thinking is [always] the attainment of some other subjective part than the one from which we have just been dislodged. It is very difficult, introspectively, to see the transitive parts as what they really are. If they are but flights to a conclusion, stopping them to look at them before the conclusion is reached is really annihilating them. Whilst if we wait till the conclusion be reached, it so exceeds them in vigor and stability that it quite eclipses and swallows them up in its glare. Let anyone try to cut a thought across in the middle and get a look at its section, and he will see how difficult the introspective observation of the transitive acts is. The results of this introspective difficulty are baleful. If to hold fast and observe the transitive parts of thought’s stream be so hard, then the great blunder to which all schools are liable must be the failure to register them, and the undue emphasizing of the more substantive parts of the stream” (James, 1890, p. 243), quoted in Atmanspacher and Fach, 2019.

This can be illustrated, for example, with the following metaphorical picture: If you cut a piece of marble in half and only look at the cut surface, observing that “the cut surface looks like this or like that,” you may have just thought of the image of the cut surface from the third person perspective. You have no way of indicating what pattern the colors and lines might follow inside the depth of the stone. In contrast to this latter stance, the cut surface is not an end in itself; it is just a means for further departure. Seeing its patterns and enormous variety of strange arrangements of colors and lines, of which it is impossible to determine the intricate ways in which they continue into the depth of the stone, its surface can be called “the appearance of one end of the cut surface (of the piece of marble or other objects).” An important point here is admitting that by merely observing the surface, you are not able to grasp its inner pattern to recognize the object structures in it. However, instead of merely stating the surface as a sober observer, you can look at it, from the stance of the introspective observer, according to James, just as you do with dream images, hypnotic phenomena, meditative states, psychedelic, drug-induced experiences (e.g., lysergic acid diethylamide, psilocybin), psychoses, and mystical experiences. First and foremost, the process described here takes place in modern and contemporary art; the mind and body can be connected by the human being and all aspects can disclose together and become visible at the same time, from cubism to abstractionism, in painting and sculpture. You can ask yourself what feelings looking at it evoke in you. Thus, you allow this aspect to be experienced as something dynamic, elusive, or intangible (e.g., *“slowing down of time and expansion of space”*).

However, the difference between viewing a sculpture, painting, cut-marble surface, etc. and the therapy process is the following: The experience of viewing art does not necessarily include the personal context (depending on the viewer’s engagement), so it may be perceived as “stochastic.” In contrast, the latter should take place within the context of the symptom. In other words, the symbol of the symptom acts as a unifying bracket that undermines the separation into stochastic events and initiates the therapeutically effective transitive state.

By returning to the clinical context, we make this process of departing start from an “aspect” possible by asking what is currently happening while simultaneously noticing subtle changes in the patient’s posture and/or minimal gestures. In this way, we encounter the instability of the patient’s current process (*“slowing down of time and expansion of space”*), which is very similar to Gendlin’s “Thinking at the Edge” (Krycka, 2006). Experience of this has been shown to trigger the process of escaping from a “stuck-state.” A quote from Gendlin from a conversation with Krycka best describes the phenomenon:

“Real thinking is rooted, emerging from ‘there’ in me. It’s an exciting, windy place where all the concepts are all trying to let energize something ‘there’—that’s the edge and we like it! We like to think. It’s just that we learned it backwards” (Personal communication of Gendlin with Krycka, May 1, 2005, quoted in Krycka, 2006).

From a neural perspective, Northoff has identified the resting-state activity of cortical midline structures as constructing the “transitive parts” and ultimately the stream of consciousness in inner time-consciousness, enabling “mental time travel” (Northoff, 2014). In their recent conceptual analysis, Northoff and Scalabrini (2021) suggested an elaborate perspective on how to utilize interactively shared space and timescales by introducing the psychotherapeutic concept of “Spatiotemporal Psychotherapy.”

“Timing, spatialness, and temporal dynamic within the interaction of client and therapist will be the key in such psychotherapeutic regulatory approach … [The] therapists [are suggested] to work using these spatiotemporal coordinates beyond the contents and the narratives of the patients. The shared time and space between therapist and client might here be seen as an operating commonly shared interpersonal spatiotemporal field, which makes possible the re-organization and transformation of the client’s intra-personal nested hierarchy of self through its spatiotemporal manifestation within her/his brain” (Northoff and Scalabrini, 2021).

Their description of working “beyond the contents and narratives” is like the “escape” process triggered by experiencing the felt meaning of the symptom, exemplified here by hypnotic trance (*time distortion in hypnosis*, according to Peter, 2015, reviewed in Häuser et al., 2016).

## Deductions from theory for clinical predictions

Depending on the clinical problem and careful recording of the patient’s history to review the symptom-inducing context, one can start with the physical or mental aspect. For example, the therapist can invite the patient to remember recently experienced pain, asking where in the body these feelings are located. Once the feeling has been stimulated, one can ask what it is and what happens; an answer is not imperative, because once the process is underway, it does not mean that it is important to understand it now, but only to maintain the current experience. Through asking, the therapist shows that experience-making, rather than knowing, is the crucial point here. You allow the feeling more and more space and suggest that subjective time slows down so that the corresponding experience can be expressed in its full temper and energy, the thoughts corresponding with local pain emerge and, simultaneously, depart, namely transitive momentum. In other words, the shared, referenced experience of the transitive momentum enables the patient to go further, toward the “Curie” symmetry of the person’s alignment to their sociocultural and body context. If successful, such utilization of the transitive (or introspective) momentum, induced here from the physical aspect, concomitantly correlates with the activation of its counterpart in the psychic aspect, through the emergence of an inner, mental image or thought content, or simply with the liberation from previous, unwanted emotions. Relief from departing pain ensues. That is, the patient is offered a way to autonomous behavior and thus to her dignity of “being human.” Note that only the core process of symptom relief is described here again to see the transitive process’s imminent relevance. Once again, I will stress here the importance of a good starting base, that is, careful history-taking to jointly decipher the fundamental meaning of patient’s various empirical bodily processes.

Conversely, you can start from the mental subdomain, such as the feeling of a form of fear. By prescribing this feeling and letting it be fully experienced, it can be denoted by its perceived meaning. In other words, the corresponding transitive (or introspective) domain is activated to mediate, for example, nausea, gag reflex, or pain in the head, stomach, or elsewhere. Anything that appears here has the sole purpose of providing experiences to refer to them. Although such experiences might be temporarily discomforting, there is an implicit sense of control over them. This changes the patient’s body posture and mood as an expression of altered bodily intero- and proprioceptive input into the brain (*mental time travel*, according to Northoff, 2014).

The proposed method, regardless of whether it is initiated by either physical or psychological subdomain, assumes that the patient is willing to accept the risk of gaining insights that may initially appear unpleasant.

Figuratively speaking, the patient may experience relief from suffering when ready to board the train that is leaving the station. Such a movement enables alignment to the current sociocultural and bodily context, that is, to attain the transitive process toward the experienced state within the given context; only then is freedom of action regained in this context. In this way, Aristotle’s saying *vita motu constat* (life consists of movement), quoted by Schopenhauer (1851/1977), is literally confirmed.

## Discussion

In summary, the purpose of this paper is to clarify whether symptom relief in psychosomatic patients is achieved by arriving at a new narrative or by the transition process itself, which lies between the old and new narrative. It has been shown here that symptom relief through verbal symbolization of bodily experiences during joint patient-therapist interaction follows symmetry acquisition, which is only theoretically attainable in its full dimension; it is the embedding of the patient in their sociocultural and body context, initially allegorized by the patient’s changed bodily experiences in the presence of the therapist. Structurally, this is consistent with findings in spatiotemporal neuroscience showing that the transitive parts of consciousness are constructed by the resting-state activity of cortical midline structures, thereby enabling “mental time travel” (Northoff, 2014) or “interpersonal attunement in time and alignment in space” (Northoff and Scalabrini, 2021). However, approaching substantive states, new aspects, or narratives produced as conceptual substrates of therapeutic change cannot be considered the critical element of therapy. Rather, these are viewed merely as phenomena from which further transitive (or introspective) states continually emanate, namely as functions of dynamic alignment of the patient’s experience with their ever-changing sociocultural and physical context. Thus, taking Degenaar (1979) as a starting point, who quoted Marx as saying that “man is a network of social relationships,” we can specify that man is a *dynamic* network of *constantly changing* social relationships. Implications for future research are compelling. What is the relevance of transitive states vs. substantive states for psychosomatic research and how does this change the approach to therapy or the dynamics of the therapist-patient relationship?

## Ethics statement

Ethical review and approval was not required for the study on human participants in accordance with the local legislation and institutional requirements. The patient provided her written informed consent to participate in this study.

## Author contributions

WT wrote the article and approved the submitted version.
